# Microfluidic Laser-Induced Nucleation of Iron (II,III) Oxide Nanoparticle-Doped Supersaturated Aqueous KCl Solutions

**DOI:** 10.1021/acs.cgd.4c00885

**Published:** 2024-09-28

**Authors:** Kelechi F. Ndukwe-Ajala, Jasmin M. Sabirin, Bruce A. Garetz, Ryan L. Hartman

**Affiliations:** Department of Chemical and Biomolecular Engineering, NYU Tandon School of Engineering, Brooklyn, New York 11201, United States

## Abstract

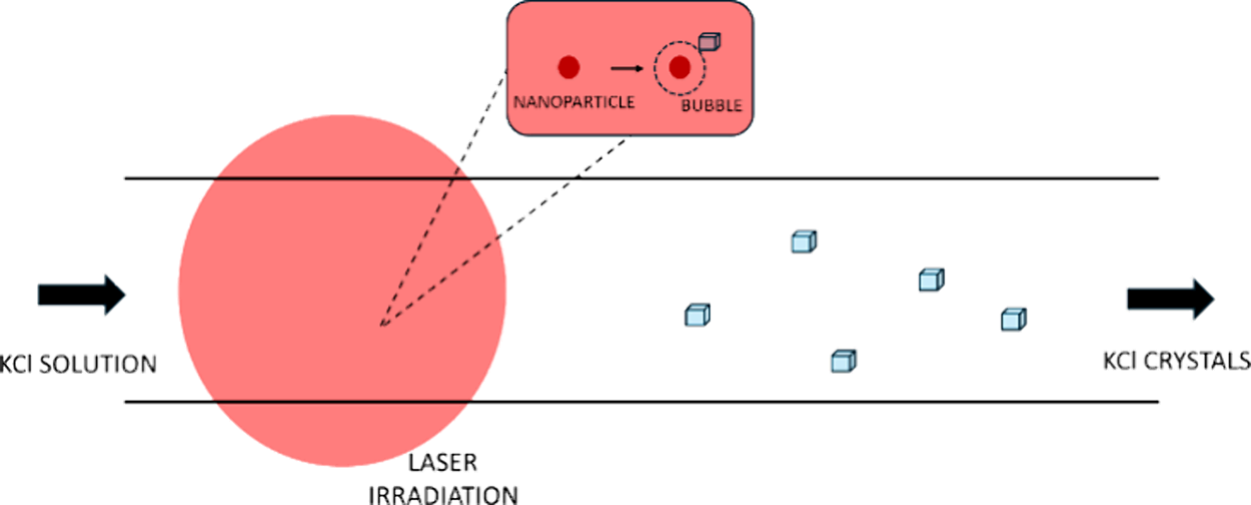

A capillary-based microfluidic system designed for nonphotochemical laser-induced nucleation (NPLIN) studies coupled with real-time microscopy was used to study NPLIN of iron (II,III) oxide doped aqueous KCl solutions. Supersaturation was achieved by lowering the solution temperature using thermoelectric cooling, and heating was used for the dissolution of crystals downstream to prevent clogging during the flow. The effect of nanoparticle concentration, supersaturation, laser intensity, and filtration was studied. We report laser-induced nucleation using laser intensities as low as 1 MW/cm^2^ with nanoparticle number densities of ∼10^9^ particles per mL of solution at KCl supersaturations from 1.06 to 1.08. The number of crystals increased with increasing laser intensity, supersaturation, and nanoparticle concentration. We discuss our results with respect to the colloidal impurity-heating mechanism hypothesis and propose a semiempirical model based on the nanoparticle heating and bubble formation due to the absorption of laser energy.

## Abstract 2

The authors studied nonphotochemical laser-induced nucleation of iron (II,III) oxide nanoparticle doped supersaturated aqueous potassium chloride solutions using a 1064 nm wavelength pulsed laser in single-phase flow. Crystal number and size were characterized in situ with real-time microscopy in the microfluidic system. A semiempirical model was developed to explain the results with respect to the colloidal impurity-heating mechanism.

## 1 Introduction

Crystallization, as a unit process, is an important step in the purification and separation of active pharmaceutical ingredients and fine chemicals. More precise control over the parameters for a desired crystallization operation would prove to be very valuable for the pharmaceutical and fine chemicals industries.^1^ Controlling crystallization using light fields for the fine chemicals industry could be a more sustainable unit process. Current industry crystallization processes utilize large amounts of chemical solvents and consume large amounts of energy during cooling crystallization. Laser-induced nucleation is poised to be a greener crystallization technique where concentrated liquid solutions containing the target solute under supersaturated conditions are exposed to the laser and crystals are nucleated; this process could be repeated wherein the solution is recycled until an optimum yield of crystals is obtained.

For wavelengths in the visible and near-infrared region with unfocused nanosecond pulses, it has been demonstrated that the nucleation rates of solutes can be increased by orders of magnitude in the presence of light. This effect, referred to as nonphotochemical laser-induced nucleation (NPLIN), offers spatial and temporal control of nucleation. The solute and solvent are exposed to the laser and are not photochemically modified owing to the negligible absorption of light at the incident wavelength. NPLIN has been observed in different solute–solvent systems: aqueous systems such as urea,^2,3^ glycine,^4−8^l-histidine,^9^ alkali halides,^10−13^ sulfates,^14^ and nitrates,^15^ as well as pharmaceutical compounds such as carbamazepine^16^ and sulfathiazole^17^ in mixed and organic solvents.

Three mechanisms have been proposed: the Optical Kerr effect (OKE) (electric field aligns disordered clusters), the Dielectric Polarization (DP) model (electric field lowers energy of subcritical clusters) and the Colloidal Impurity-heating (CI) mechanism (nanobubble formation around heated nanoparticles induces nucleation). The OKE model was initially proposed to explain the experimental observations from the first system studied of aqueous urea solutions.^2^ According to this model, in the presence of the laser, molecules in the precritical clusters align in the direction of the laser beam’s electric field; however, Monte Carlo simulations showed that the theoretical electric field strength needed to lower the nucleation barrier sufficiently is orders of magnitude higher than that used in experiments.^8^ The DP model was proposed to fit the crystal yield versus laser intensity in the laser-induced nucleation of aqueous potassium chloride solutions.^18^ The nucleation barrier of subcritical clusters is lowered in the presence of the laser owing to the difference in dielectric constant between the solute clusters and the surrounding solution. This model quantitatively predicted a linear dependence of the number of crystals formed on the laser intensity, although the model had to be corrected for a nonzero laser intensity threshold observed during experiments. However, the model could not explain NPLIN of CO_2_ bubbles in aqueous solution where the gas phase has a lower dielectric constant than water. Moreover, neither the DP nor OKE model could explain the reduced nucleation probability with filtration or subsequent increase with intentional doping with impurities. The CI mechanism posits that nanoimpurities present in the solution are heated up due to the absorption of laser energy; the heated particle vaporizes the surrounding solvent thus forming a nanobubble around which crystallization is induced at the vapor–liquid interface. Moreover, many features of NPLIN experiments, such as the effect of filtration and laser intensity threshold, could be explained by the CI mechanism.^19^ It also could be possible that a combination of these mechanisms might explain NPLIN.

The majority of laser-induced nucleation studies have been performed in glass vials (batch method), but microfluidics offers a better alternative experimental technique for crystallization experiments. Microfluidic systems offer ideal heat and mass transfer at micrometer length scales, isolation from contaminants, reduced material consumption and high-throughput in situ characterization methods.^20^ Localized temperature control of the microfluidic system^21,22^ provides the opportunity to induce supersaturation during flow and prevents the need for preparation and storage of large numbers of glass vials or batch reactors. Previously in our group, a continuous single-phase microfluidic setup utilizing a sandwiched glass-polydimethylsiloxane-glass microfluidic chip was used to explore NPLIN for aqueous potassium chloride solutions^10^ and aqueous glycine solutions^4^ to observe the influence of laser intensity, laser exposure time, flow rate, and supersaturation on the crystal yield.

Studies exploring the role of impurities in laser-induced nucleation have supported the CI mechanism. Knott et al.^8^ observed NPLIN of CO_2_ gas in water where the dielectric constant of the solute is less than that of the solvent which ruled out the DP mechanism. Javid et al.^5^ found that nanofiltration (0.2 μm pore filter) of glycine solutions prior to irradiation significantly increased the observed induction times and reduced the percentage of crystallized samples by 80% compared to nonfiltered samples, suggesting that nanofiltration removed colloidal-scale solute clusters, and/or colloidal impurity particles. Ward et al. analyzed the impurities present in unfiltered ammonium chloride solutions and found iron to be a major component; by intentionally doping filtered aqueous ammonium chloride solutions with iron (II,III) oxide nanoparticles, the nucleation probability increased to prefiltered levels.^23^ The term “doping” as used here and in other NPLIN studies,^23,24^ refers to the intentional addition and dispersal of nanoparticles in liquid solutions. From observations of NPLIN in aqueous CO_2_ solutions, a simple model was developed that describes an impurity nanoparticle heated by the laser pulse causing a water vapor bubble to form around it; this vapor cavity acts as a seed for an influx of CO_2_ and subsequent growth of a carbon dioxide gas bubble.^25^ Additionally, the nonzero laser intensity threshold common in previous studies might be as a result of the effect of the nature and varying concentrations of impurities in samples tested. Kacker et al.^11^ observed nucleation at intensities as low as 0.5 MW/cm^2^ for unfiltered aqueous potassium chloride and 3.0 MW/cm^2^ for 450 nm filtered solutions at a fixed supersaturation of *S* = 1.049 with a 1064 nm laser, which suggests the role of impurities on the laser intensity threshold for NPLIN systems. Atomistic molecular dynamic simulations, which tested the effect of a heated 2 nm nanoparticle on the structure of a supersaturated aqueous sodium chloride solution, showed that the local salt concentration was strongly depleted 1.5–2.5 nm from the particle surface over 2–3 ns.^26^ This suggests that an increased clustering of ions occurs arising from the nanoparticle-heating. At a fixed laser intensity of 12 MW/cm^2^, Ward et al.^23^ showed that filtering or long-term exposure to laser pulses reduced the crystal yield, which could be returned to unfiltered levels by intentionally doping with iron (II,III) oxide nanoparticles of ∼4 × 10^11^ nanoparticles per mL solution.

Korede et al.^24^ have reported laser-induced nucleation of aqueous potassium chloride solutions with iron (II,III) oxide nanoparticles using a two-phase microfluidic system. Aqueous KCl droplets were dispersed in a second continuous phase consisting of silicone oil. Each droplet acted as a separate reactor, and generated results from over 1000 droplets for each parameter investigated. Solutions with the lower supersaturation (*S* = 1.05) exhibited a threshold intensity of 50 MW/cm^2^ and the higher supersaturation (*S* = 1.10) showed a threshold intensity of 10 MW/cm^2^.

In this work, we report laser-induced nucleation of iron (II,III) oxide doped aqueous potassium chloride solutions at laser intensities down to 1 MW/cm^2^ with nanoparticle concentrations of ∼10^9^ nanoparticles per mL of solution at supersaturations from 1.06 to 1.08 using a single-phase microfluidic system. The samples used in this work were nanofiltered with a 20 nm pore size prior to the addition of nanoparticles. The continuous process of injection, supersaturation, laser-induced nucleation, and real-time microscopy ensured a faster screening of experimental parameters and data collection. We report crystal yield versus laser intensity for intentionally doped aqueous KCl solutions and propose a semiempirical model based on nanoparticle heating due to the absorption of laser energy to explain the results.

## 2 Experimental Methods

### 2.1 Materials

Potassium Chloride (99% ACS grade, VWR) was dissolved in ultrapure 0.22 μm filtered deionized water from a Milli-Q system (resistivity 18.2 MΩ cm, Millipore) in Schott glass bottles. Iron (II,III) oxide nanoparticles dispersed in water (Particle diameter = 30 ± 3 nm (TEM) and concentration of 5 mg/mL, Sigma-Aldrich) was used to intentionally dope the aqueous potassium chloride solutions.

### 2.2 Solution Preparation

All glass bottles and screw caps were washed thoroughly with soap solution (Micro-90) and rinsed at least 3 times with about 500 mL of ultrapure deionized water and dried before use for solution preparation. Slightly undersaturated aqueous KCl solutions at 20 °C were prepared for this study. The solubility of KCl in water is 0.3436 g per 1 g water at 20 °C.^27^ Supersaturation here is defined as *S* =  where *c* = solution concentration and *c*_sat_ = concentration of a saturated solution at a given temperature. Supersaturations defined in this study are with respect to the saturated concentration at the set cooled zone temperature. A solution concentration of 3.89 M, which corresponds to 0.341 g KCl per 1 g water, was prepared by dissolution of solid potassium chloride in ultrapure deionized water in clean Schott glass bottles within a dust-free environment. A magnetic stirrer bar was added for mixing; then the glass bottle was placed on a hot plate stirrer at 45 °C. After dissolution for 1 h, the glass bottle was transferred to an incubator oven and kept overnight at 45 °C before use.

### 2.3 Solutions Doped with Nanoparticles

Solutions as prepared above were doped with iron (II,III) oxide nanoparticles. Warm KCl solutions were filtered with 20 nm pore size syringe filters, which have an alumina-based membrane in a polypropylene housing (WHA68091002, Whatman Anotop), as they were transferred into warm clean glass bottles. Glass bottles, caps, syringes, needles, and syringe filters used were kept in the incubator oven to warm up prior to use. A known volume (∼120 μL) of iron oxide nanoparticle dispersion was carefully added to the glass bottle containing the filtered 3.89 M KCl solution to achieve a desired nanoparticle number density and the desired KCl concentration of 3.88 M which corresponds to 0.34 g KCl per 1 g water. The procedure to prepare the stock Fe_3_O_4_ dispersion used to dope KCl solutions and calculations for the nanoparticle concentration are detailed in Section S.2. The glass bottle was ultrasonicated in an ultrasonic cleaner (VWR) for 1 h at room temperature to ensure optimal dispersion of the nanoparticles in solution and was allowed to sit for 30 min before use in experiments. The solution was inspected by eye to ensure that no nucleation occurred prior to use for experiments. Doped solutions were left out at room temperature for 4 weeks, and no spontaneous nucleation was observed.

### 2.4 NPLIN Flow Experiment

The microfluidic setup utilizes a piston reciprocating pump (LS005SRX, Teledyne Instruments) to deliver the solution at a constant flow rate of 300 μL/min. A 1 × 1 mm square ID, 0.2 mm wall thickness and 100 mm long glass capillary (8100, VitroCom) was connected using clear epoxy (EA E-05CL, Loctite) to standard cylindrical PEEK tubing using in-house 3D printed (Form 3 printer) connector sleeves. The square glass capillary was chosen to provide an optimal flat field during viewing with the microscope. The square side walls also eliminate any laser focusing such as occurs with cylindrical vials. Connector sleeves with a square port on one end and circular port on the other were designed and 3-D printed to address issues of unwanted spontaneous crystallization at connection points. These sleeves were made of clear polycarbonate resin with a layer resolution of 25 μm, which ensured a relatively smooth surface to prevent heterogeneous nucleation.

**Figure 1:**
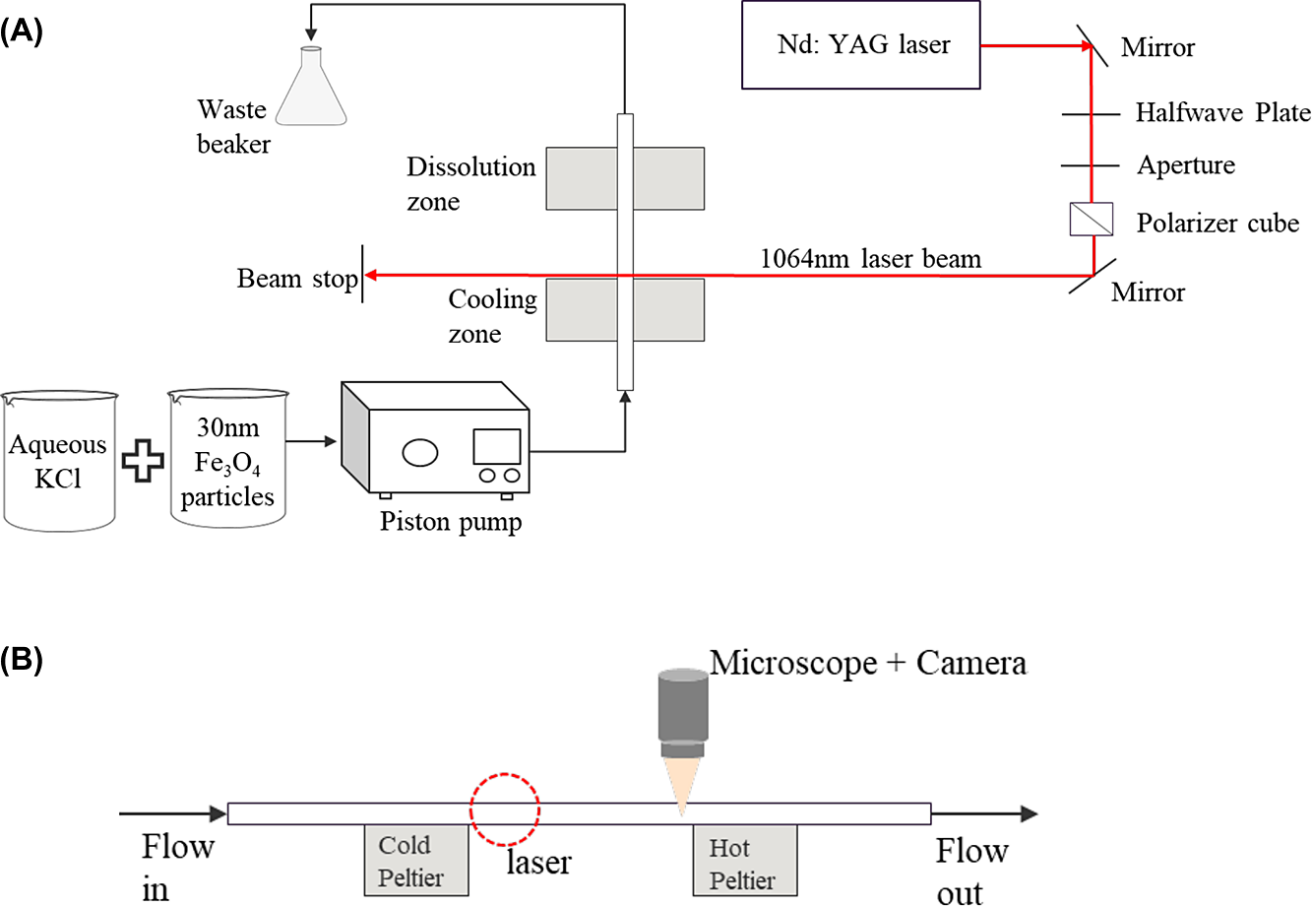
(A) Flow diagram of the experiment setup. (B) Side view of the flow path showing laser position and observation location.

On the left side of Figure 1A, the flow path of the solution is described. The solution is injected into the capillary using the piston pump, interacts with the laser in the irradiation zone, exits the capillary and is collected in a waste beaker. The capillary is in contact with two thermoelectric Peltier coolers of dimensions 20 × 40 × 4 mm (TE-63-1.4-1.5, TE Technology) using thermal paste (52022JS, AOS) to ensure continuous contact between the Peltier surfaces and the glass capillary as well as to provide uniform heat transfer at both cooling and dissolution zones. An aluminum block heat exchanger placed under the Peltier coolers was used to circulate water at 20 °C to continuously remove heat generated on the hot side of the cold Peltier. Both thermoelectric devices were independently controlled with PID controllers (TC-48-20, TE Technology), and the Peltier surface temperatures were monitored using thermistors (TE Technology MP-2444); temperature fluctuations recorded were <0.3 °C. The hot Peltier was set at 45 °C for all experiments.

Supersaturation was induced by local subcooling of a section of the capillary. The temperature required for an induced supersaturation value was estimated using published solubility data,^27^ which was the same approach used by Hua et al.^10^ (Figure S.1 in Supporting Information). The induced supersaturation was achieved before the KCl solution interacted with the laser in the irradiation zone owing to the fast conductive heat transfer at reduced length scales offered by microfluidics.

A continuous train of laser pulses (1064 nm wavelength, 6 ns duration) was generated by a Q-switched Nd:YAG laser (Continuum, Surelite II-10). The wavelength used was chosen to compare results from this study with previous studies on laser-induced nucleation of potassium chloride,^10^ and it has been observed that there is a weak dependence of nucleation probability on the wavelength.^11^ The pulse repetition rate was set at 10 pps. As shown on the right side of Figure 1B, the 7 mm diameter laser beam produced from the laser was directed by a mirror and then through a zero-order halfwave plate. A 5 mm diameter ceramic aperture was placed in the beam path to reduce the beam diameter accordingly. The incident laser beam was linearly polarized using a Glan Taylor Calcite Polarizer cube (10GL08AR.33, Newport Corp.). The beam was directed by a pair of mirrors upward and toward the glass capillary and stopped on the opposite side using a metal beam stop. The average laser power incident on the glass capillary was measured with a thermopile sensor (PM30V1, Coherent) connected to a power meter (LM30V, Coherent). The laser power could be varied by either rotating the halfwave plate or changing the Q-switch delay; for convenience, we used the Q-switch delay.

To capture images and videos of KCl crystals in flow, a 20MP CMOS camera (MU2003-BI, Amscope) mounted on a metallurgical microscope (ME580TC-PZ-2L, Amscope) with a 10x objective (Amscope 10X/0.25 Plan Achromatic) provided a resolution of 1.37 pixels per micron. Crystals were observed about 7.5 mm downstream from the center of the irradiation zone in the glass capillary. For all acquisitions, the camera was focused at a depth of ∼80 μm above the internal capillary floor and captured videos at 60 fps with an image size of 1824 × 1216 pixels.

### 2.5 Dynamic Light Scattering/Zeta Potential Experiment

Dynamic light scattering experiments were conducted using a Zetasizer Nano-ZS (Malvern Panalytical) with a 633 nm laser and a backscattering angle of 173°. Solution samples tested were prepared in the same manner as those used for NPLIN experiments. Pipette tips and disposable cuvettes (DTS0012, Malvern Panalytical) were preheated at 45 °C to avoid spontaneous nucleation during transfer. The solution was carefully transferred into cuvettes while hot, then allowed to cool down to room temperature. Cuvettes were equilibrated in the DLS instrument at 25 °C for 2 min before measurement. The instrument averaged 11 scans in one run and each run was repeated 3 times. At 25 °C, the estimated supersaturation is *S* = 0.95, and in addition to this undersaturated state, the weak power (<10 mW) of the continuous wave laser used in the instrument is not expected to trigger nucleation during measurements.

For Zeta Potential experiments, special cuvettes (DTS1070, Malvern Panalytical) were used. A well-mixed suspension of 500 μL of 0.17 mg/mL 30 nm Fe_3_O_4_ nanoparticles in water and 500 μL of 10 mM aqueous KCl, which corresponds to 1.17 × 10^12^ nanoparticles/mL, was tested. Each run was repeated 3 times. A higher nanoparticle concentration was used to get reliable results with the instrument as the concentrations used in this NPLIN study produced irreproducible zeta peaks.

## 3 Results

**Figure 2:**
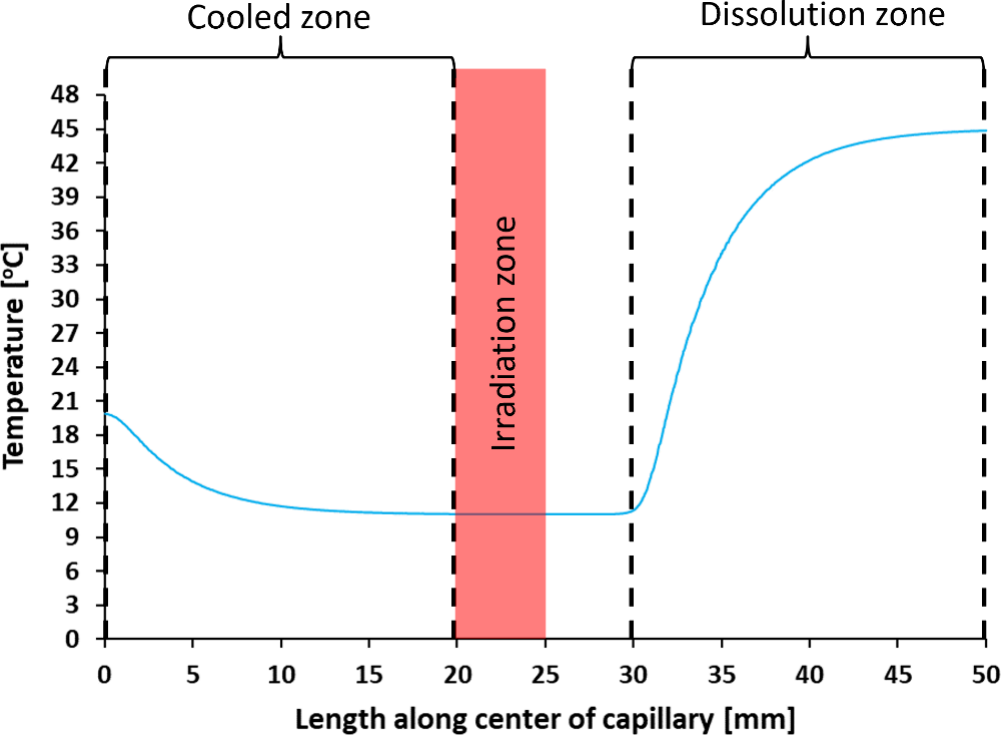
CFD simulation result of temperature profile along the capillary length in the center of the channel with KCl solution flowing at 300 μL/min where the cooled zone is at 11.0 ± 0.2 °C and the dissolution zone is at 45 ± 0.3 °C.

Computational fluid dynamics (CFD) simulations were performed for the capillary setup to determine if the target supersaturation could be induced before the solution entered the irradiation zone of the capillary. Details about the CFD simulations and infrared camera images are provided in Section S.3. Simulation results agree with infrared camera images that the solution reaches the set cooled zone temperature at the irradiation zone (Figure 2).

**Figure 3:**
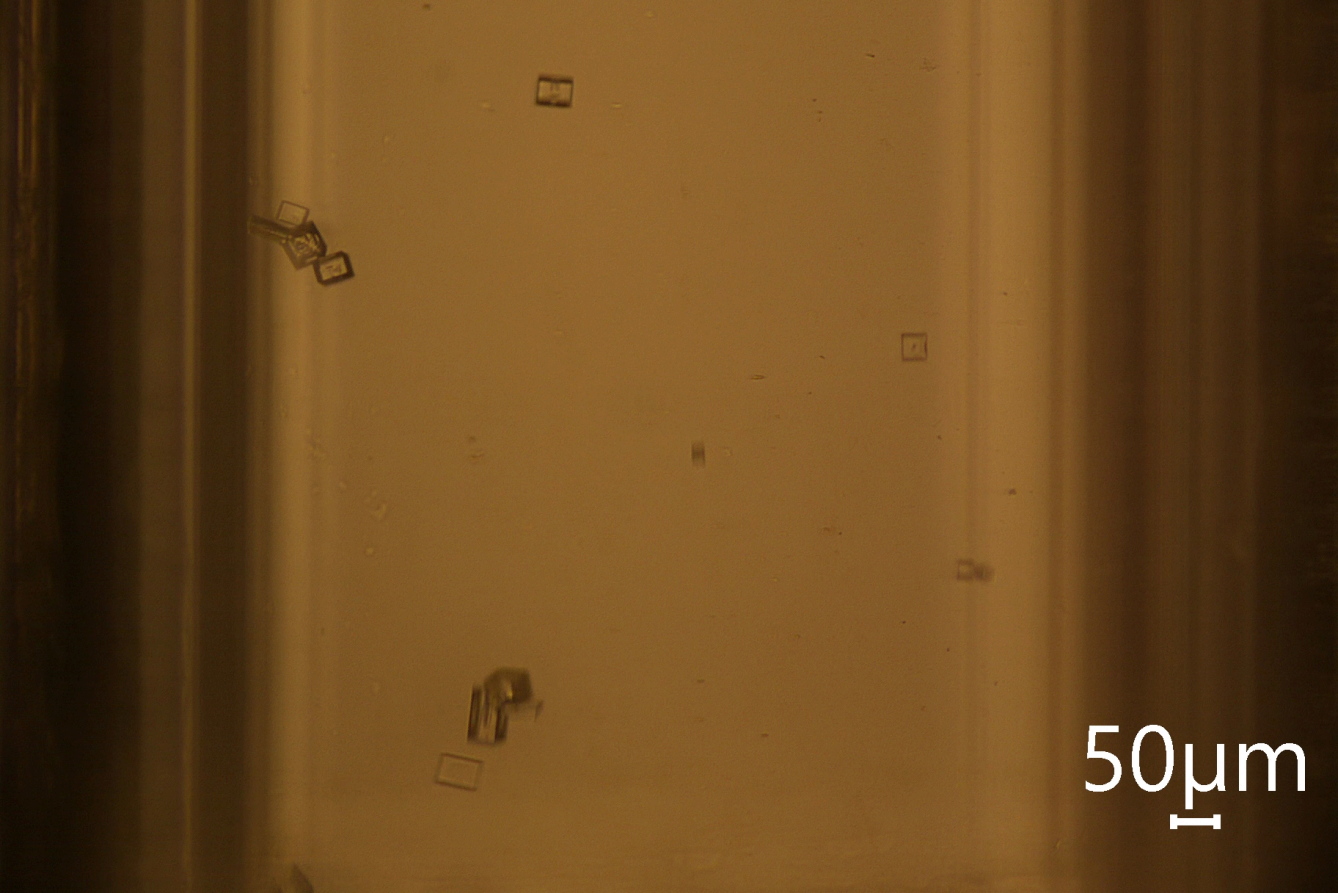
Snapshot of KCl crystals captured flowing through the capillary at 300 μL/min.

Control experiments were conducted in which the solution volumes were run through the capillary with supersaturation induced locally, but not exposed to the laser. These control experiments were performed for every parameter set tested in this study for at least 5 min prior to tests with the laser turned on. No trace of nucleation/crystallization was observed indicating that heterogeneous/spontaneous nucleation was not occurring and that a consistent zero-crystal baseline can be assumed at zero laser intensity (Figure 3).

The volume of the irradiation zone was 5 μL. At a flow rate of 300 μL/min, the residence time of a solution volume element in the irradiation zone was 1 s. At a laser repetition rate of 10pps, each volume element was exposed to 10 laser pulses on average. The total capillary volume is 100 μL, and at a flow rate of 300 μL/min, the residence time of solution in the capillary was 20 s. The solution was allowed to flow for a minimum of 5 min to ensure that a steady state of flow was reached. By varying the experiment run time, a manageable number of crystals could be observed while avoiding the clogging of the capillary channel due to crystal buildup at the outlet end. A compilation of the crystal yield per experiment is included in Section S.4. Real-time microscopy enabled the capture of crystal images in the capillary while in flow. Microscopy software accompanying the Amscope camera was used to count the number of crystal images captured and measure the crystal dimensions.

### 3.1 Effect of Filtration

**Figure 4:**
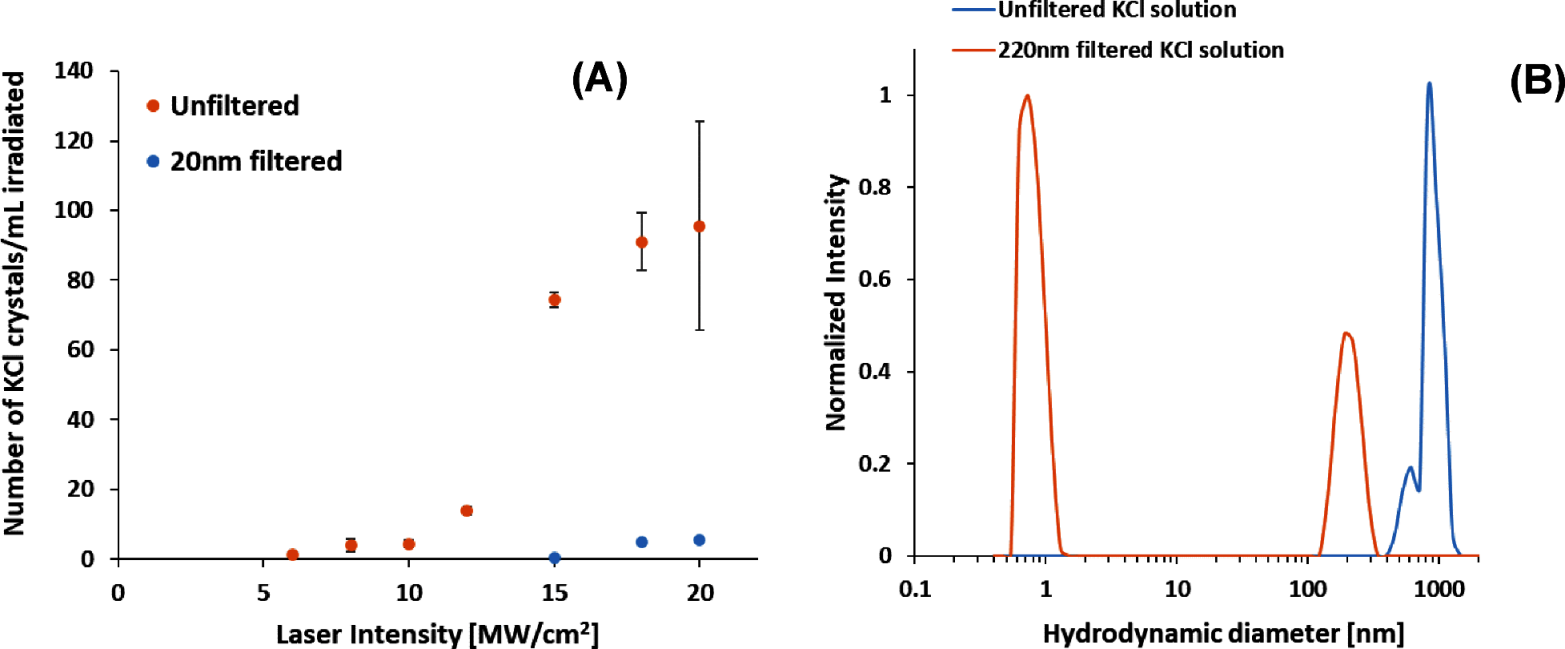
(A) Effect of Filtration on the NPLIN of aqueous KCl at a supersaturation of 1.06. Error bars represent standard deviations from averaging the results of three runs at each intensity. (B) DLS results for unfiltered and 220 nm filtered KCl solutions.

Solutions that were not filtered after solution preparation are considered here as unfiltered. In addition, we note that all solutions were prepared carefully to avoid dust particles. Filtered solutions were prepared using a 20 nm syringe filter. Crystal yield numbers for 20 nm filtered solutions were an order of magnitude lower than those of the unfiltered solutions (Figure 4); for instance, at 20 MW/cm^2^ and *S* = 1.06 about 5 crystals/mL nucleated compared to ∼100 crystals/mL nucleated from unfiltered solutions under the same conditions. This reduction in crystal yield with filtration is consistent with other published observations and provided a baseline to observe NPLIN for doped solutions. Dynamic Light Scattering results also showed the presence of particles about 900 nm in size in the unfiltered solution and about 200 nm particles in the 220 nm filtered solution. Particles <1 nm have been attributed to solute scattering.^23^ The particles present in the 20 nm filtered solutions were below the detectable number concentration limits of the DLS instrument. Ward et al.^28^ performed quantitative particle tracking using a custom in-house setup on a filtered (220 nm filter pore size) ammonium nitrate solution and observed a particle number density of ∼2 × 10^6^ cm^–3^, while a commercial nanoparticle tracking analysis (NTA) instrument (Nanosight, LM10) detected no particles due to the limit of resolution of the instrument (*d* ≤ 20 nm).

### 3.2 Nanoparticle Characterization

**Figure 5:**
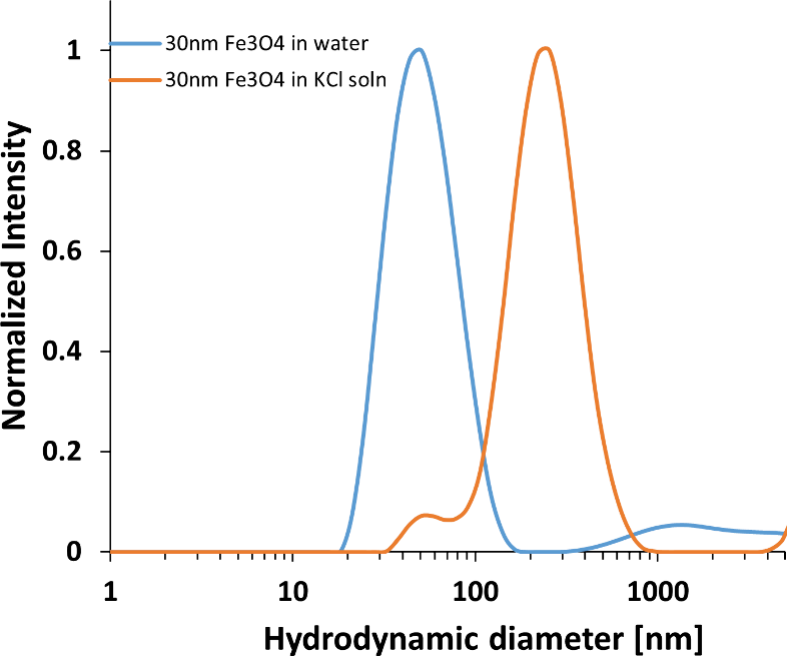
Plot showing particle size distributions obtained for iron oxide nanoparticles in water and aqueous KCl solution.

After conducting mass spectrometry analysis which indicated the presence of iron impurities and which was consistent with the solute manufacturer’s purity analysis, Ward et al.^23^ intentionally doped aqueous ammonium chloride solutions with Fe_3_O_4_ nanoparticles. Solutions in our study were doped with 30 nm Fe_3_O_4_ nanoparticles in a similar fashion.^23,24^ Ward et al.^23^ used 20–30 nm particles, and Korede et al.^24^ used 50–100 nm particles. In our DLS experiments, for the nanoparticles dispersed in water, the mean hydrodynamic diameter detected was 52 nm, but when these nanoparticles were dispersed in KCl solution, DLS detected species present with a mean hydrodynamic diameter of 238 nm (Figure 5); careful and clean preparation of the samples was carried out to minimize dust particles, and species typically of a few microns in size were attributed to dust particles and tended to settle due to density differences compared to water. Detected species of about 200 nm in diameter suggest that the 30 nm particles are possibly interacting strongly with a surrounding shell of ions in the solution.^23^ These interactions could be electrostatic considering the ligands used to disperse the particles in an aqueous medium. For these particles, the surface is functionalized with carboxyl groups that can provide a net negative charge to the nanoparticles to help them repel each other; this ensures a well dispersed and stable suspension. Zeta Potential measurements of the iron oxide nanoparticles in aqueous KCl solution yielded a zeta potential of −36.9 ± 1.5 mV which indicates a net negative surface charge on the nanoparticle. A common guideline for colloidal stability is that a magnitude >30 mV indicates a highly stable colloidal suspension.^29^ This implies that sufficient repulsion of the particles with themselves prevent clustering, but in addition the negatively charged surface likely facilitates a strong interaction with the potassium ions in the aqueous KCl solution.

### 3.3 NPLIN in Doped Solutions

**Figure 6:**
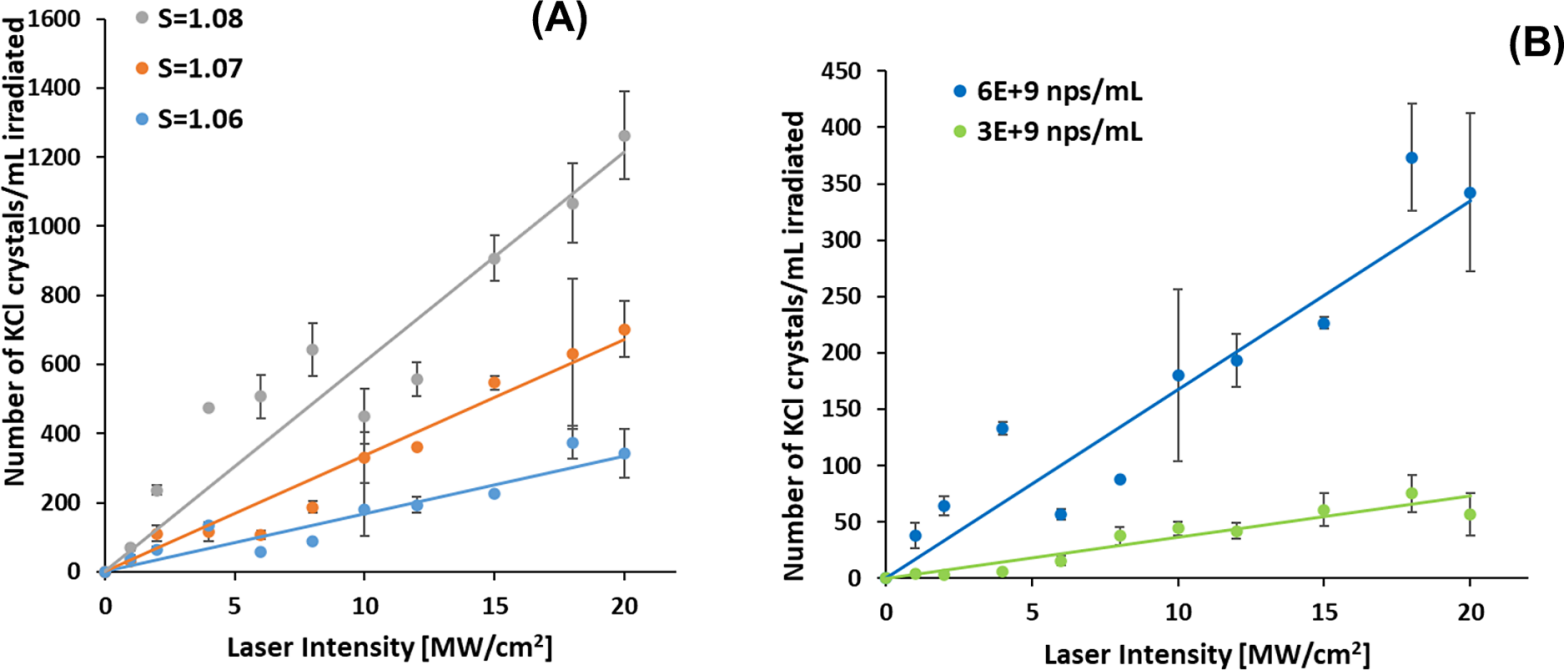
Plots of *N*_crystal_ vs laser intensity, showing: (A) Effect of supersaturation at a nanoparticle number density of 6 × 10^9^ nanoparticles per mL doped KCl solution. (B) Effect of nanoparticle number density at a fixed supersaturation of *S* = 1.06. Error bars represent standard deviations from averaging the results of three runs at each intensity.

Figure 6 displays the number of crystals observed as a function of laser intensity for 3 different KCl supersaturations and 2 different Fe_3_O_4_ nanoparticle concentrations. These experiments produce larger numbers of crystals compared to those of Hua et al.^10^ For example, at *S* = 1.06 with 6 × 10^9^ nanoparticles/mL and laser intensity of 20 MW/cm^2^, we observe 340 KCl crystals per mL of irradiated volume, whereas Hua et al. reported about 1.5 crystals per mL of irradiated volume for a 220 nm filtered solution at the same supersaturation and laser intensity. We attribute the difference in the crystal yield to the added dopant used and its concentration.

The experimental data were well approximated by a linear fit of the form:
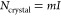
1where *N*_crystal_ is the number of crystals observed per mL of irradiated solution, *I* is the laser intensity, and *m* is the lability. The lability is a measure of how likely the sample is to nucleate; the higher the lability means more nucleation events at a given laser intensity. The term “lability” was first introduced by Alexander and Camp^18^ in the context of the NPLIN of aqueous KCl, and it was related to parameters of the DP model. In this paper, we will relate the lability to parameters of the CI model. A linear regression fit with a zero intercept was applied to the data sets. If there is an intensity threshold for nucleation, it is below the lowest intensity used in our experiments (1 MW/cm^2^); this low intensity threshold may be a consequence of the high absorption efficiency and high concentration of the Fe_3_O_4_ nanoparticles used in this study. As shown in Figure 6A as the supersaturation increases, the lability increases, consistent with previous studies. The lability also increases as the nanoparticle concentration increases, as shown in Figure 6B.

**Figure 7:**
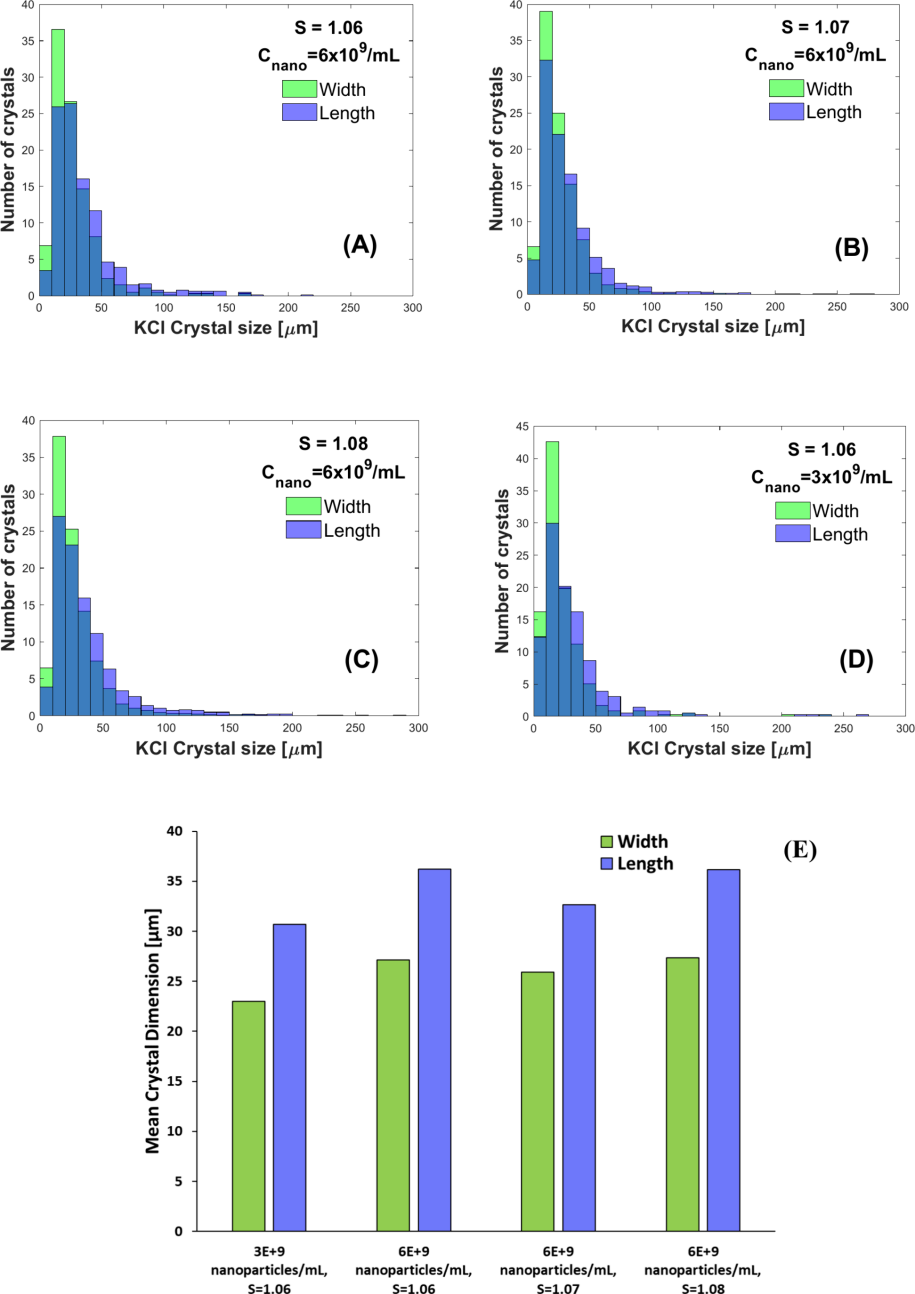
KCl crystal size distribution plots for results of 6 × 10^9^ nanoparticles/mL solution at (A) *S* = 1.06, (B) *S* = 1.07, (C) *S* = 1.08, (D) 3 × 10^9^ nanoparticles/mL solution at *S* = 1.06, and (E) the mean crystal dimensions for all experimental data sets.

The crystal size distribution data for our experiments exhibit a normal distribution with a mode of about 30 μm for all supersaturations and nanoparticle concentrations as shown in Figure 7. At a nanoparticle concentration of 6 × 10^9^ nanoparticles/mL and for the supersaturations tested, we calculated the mean crystal width to be 27, 26, 27 μm and the mean crystal length to be 36, 32, 36 μm at *S* = 1.06, 1.07, 1.08 respectively. For 3 × 10^9^ nanoparticles/mL at *S* = 1.06, the mean crystal width and length were 23 and 30 μm, respectively. Given the 7.5 mm distance between the center of the irradiation zone and the observation point downstream of the capillary, we can estimate that a crystal moving at 300 μL/min will take 1.5 s to move through that distance. Assuming a critical nucleus radius^30^ of 2.8 nm for KCl at *S* = 1.06, we can estimate a linear growth rate: Δ*L*/Δ*t* = (30 μm–2.8 nm)/1.5 s ≈ 20 μm/s.

### 3.4 Laser Heating of Nanoparticles

In the present work, the experimental results substantiate the claim that the nanoparticles play a major role in facilitating the laser-induced nucleation of potassium chloride crystals. In an effort to explain the results in Figure 6, within the framework of the CI mechanism, we hypothesize a sequence of events that leads to nucleation of potassium chloride crystals:

1. Light is absorbed by the nanoparticle
2. The absorbed energy is rapidly transferred as heat to the surrounding liquid
3. A surrounding shell of the solvent is vaporized to produce a bubble
4. The solute molecules previously inside that surrounding shell of liquid are pushed to the bubble-solution interface, aggregate to form a critical nucleus, and grow into a macroscopic crystal.

The methodology used to estimate the size of the bubble formed is the same as the approach utilized by Ward et al.^25^ where the final bubble radius is dependent on the incident laser intensity as well as the nanoparticle’s size and absorption efficiency and the thermophysical properties of the solvent such as density and heat capacity. Calculations for the final bubble radius for a 30 nm Fe_3_O_4_ particle exposed to a laser intensity of 1 MW/cm^2^ for a 6 ns duration are detailed in Section S.5 of the Supporting Information.

If a vapor bubble concentrates solute molecules at the bubble/solution interface, then the larger the bubble, the more highly concentrated the solute molecules become at the interface, and therefore the greater the probability that a solute crystal will nucleate. For simplicity, we assume that the probability of nucleation near a heated nanoparticle is proportional to the bubble volume:
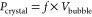
2where *f* is a proportionality factor. For experiments carried out at higher supersaturations, we would expect that the concentration of solute molecules at the interface to be even larger, so that the constant *f* is expected to increase as the supersaturation increases.

Based on eq 2, the number of crystals that are formed would be equal to the number of irradiated nanoparticles, *N*_irrad_ times *P*_crystal_:
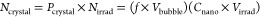
3where *C*_nano_ is the number density of nanoparticles and *V*_irrad_ = 1 mL is the total irradiated solution volume. From bubble size estimations, there is a linear dependence of the bubble volume on the laser intensity so the bubble volume can be expressed as *V*_bubble_ = *cI*, where *c* can be estimated from calculations shown in Section S.5. Thus, *N*_crystal_ can be expanded in this form:
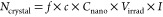
4

Utilizing the linear fit described in eq 1, *f* can be expressed in terms of the lability, *m*, as follows:
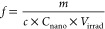
5

For a supersaturation of 1.06 and a nanoparticle concentration of 6 × 10^9^ nanoparticles/mL, the estimated lability constant from the linear fit is *m* = 16.75 cm^2^/MW. From eq 5, we calculate *f* to be 8.36 × 10^6^ mL^–1^. The fitted parameters for all conditions are listed in Table 1.

**Table 1:** Labilities, Obtained by Fitting the Experimental Data to eq 1, and Model’s Proportionality Factor, Obtained from eq 5^a^

experimental parameters	lability constant (cm^2^/MW)	*f* (10^6^ /mL)
3 × 10^9^ nanoparticles/mL, *S* = 1.06	3.65 ± 0.45	3.64 ± 0.45
6 × 10^9^ nanoparticles/mL, *S* = 1.06	16.75 ± 1.85	8.36 ± 0.93
6 × 10^9^ nanoparticles/mL, *S* = 1.07	33.55 ± 2.99	16.78 ± 1.50
6 × 10^9^ nanoparticles/mL, *S* = 1.08	60.75 ± 4.85	30.44 ± 2.43

a Uncertainties based on 95% confidence limits.

We are now in a position to calculate the probability that a heated nanoparticle of a given volume gives rise to nucleation of a potassium chloride crystal. For a supersaturation of 1.06, a nanoparticle concentration of 6 × 10^9^ mL^–1^ and a laser intensity of 20 MW/cm^2^, the probability that an irradiated nanoparticle results in a crystal forming can be estimated using eq 2 and equals 5.58 × 10^–8^ or about one in 18 million.

Our model assumes that the number of crystals that form should be proportional to the number of irradiated nanoparticles, which in turn is proportional to the nanoparticle concentration. While we do observe in Figure 6B that increasing the nanoparticle concentration increases the number of crystals observed, it does not appear to be exactly proportional. This is also reflected in the fact that the calculated *f* values at *S* = 1.06 are different for the 2 different concentrations.

## 4 Discussion

We hypothesize that the absorption of laser energy by a nanoparticle results in the heating of the nanoparticle, and that the transfer of heat to the surrounding solution vaporizes the solvent, forming a vapor bubble that expands due to explosive evaporation. The semiempirical model developed in this study assumes that the probability of nucleation, *P*, is proportional to the bubble volume, *V*_*bubble*_. The reasons for this choice are 2-fold: (1) it is simple and plausible, and (2) it results in the observed number of crystals being proportional to the laser intensity, *I*, which agrees with the experimentally observed linear dependence of *N*_crystal_ on *I*. A plausible alternative choice is to assume that *P* is proportional to the surface area of the bubble, *A*_bubble_. Owing to the fact that , this alternative choice results in the prediction that , in disagreement with our experimental observations. Hence, we can discount that choice.

While nanobubbles have not been observed in NPLIN experiments using unfocused nanosecond laser pulses, cavitation bubbles that are hundreds of micrometers in diameter have been observed in experiments^31,32^ using tightly focused nanosecond laser beams. These experiments also reveal the nucleation of small crystals near the surface of the bubbles, lending support to our proposed mechanism. In these experiments, the laser intensities are 3 orders of magnitude larger than those used in our experiments. For example, the highest intensity used in our experiments was 20 MW/cm^2^, whereas focused-beam studies^31^ used intensities as high as 30 GW/cm^2^. In those focused-laser experiments, soluble light-absorbing impurities were purposely dissolved in the solution to ensure that sufficient energy was absorbed at the focal laser spot to induce thermocavitation in the solution. Such an approach would be ineffective with unfocused laser beams because the absorption of light would be spread out over a large volume of the solution. The spatial localization of energy absorption using an unfocused beam is made possible with colloidal impurity particles that strongly absorb the incident light. Soare et al.^31^ estimated that the local solution temperature reached 494 K at the focal laser spot for laser-induced thermocavitation of aqueous ammonium sulfate. Hidman et al.^33^ performed cavitation experiments with a focused beam and predicted with simulations that the solution temperature after the laser pulse should be higher than 494 K. This would agree with a temperature of 520 K required for water nanobubble formation as assumed in the model developed in this study.

During the laser energy absorption phase by the nanoparticle, the nanoparticle temperature is estimated to reach as high as a few thousands of Kelvins.^26^ These temperatures result in explosive evaporation of the surrounding solvent around the nanoparticle. This condition may trigger chemical reactions for a complex salt solution system but for simple salts such as KCl, it is hard to imagine what photochemical species would be involved in a possible reaction as the solute is not chemically altered and the crystals nucleated were of typical morphology. Therefore, nucleation of crystals via a mechanism that generates a transient vapor bubble at a solid nanoparticle by heating after laser light absorption could still be referred to as nonphotochemical laser-induced nucleation.^23^

**Figure 8:**
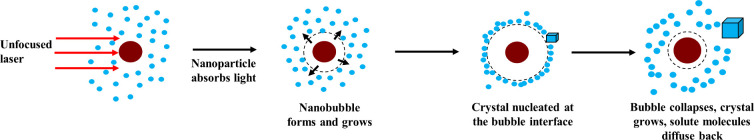
Schematic showing the sequence of events for impurity-heating mechanism. Red circle refers to the 30 nm Fe_3_O_4_ nanoparticle and the blue circles refer to the solute molecules in solution.

In Figure 8, the sequence of crystallization events is shown for a single nanoparticle. However, in this work, nanoparticles with concentrations on the order of 10^9^ per mL are exposed to the laser, so many nanoparticles are simultaneously exposed to the laser pulse. We rationalize that each nanoparticle’s interaction with the solution can be treated as independent of the others if the distance between the particles is sufficiently large for thermal interactions to be negligible. Assuming nanoparticles are equally spaced in a cubic lattice, we can estimate the spacing between particles. Estimations of the distance between nanoparticles for a density of 6 × 10^9^ nanoparticles/mL revealed that the spacing between nanoparticles is about 5.5 μm which is 2 orders of magnitude larger than the nanoparticle size. Furthermore, femtosecond laser excitation of gold nanoparticles in water shows that temperature changes do not extend farther than about 50 nm.^34^

The proposed mechanism assumes that the nanobubble formed eventually collapses as the crystal nucleus grows. In our experiments, a solution volume element is exposed to irradiation for 1 s, during which the solution is exposed to 10 laser pulses, separated in time by 0.1 s. We estimate the bubble size using a single laser pulse duration of 6 ns; it could be the case that multiple bubbles cycle through formation and collapse around the nanoparticle during the laser exposure in experiments. We can estimate the time required for a bubble to collapse within a liquid using the Besant-Rayleigh-Plesset equation.^35^ Using a bubble radius of 43 nm, as estimated for a 30 nm Fe_3_O_4_ particle exposed to a laser intensity of 1 MW/cm^2^ for a 6 ns pulse duration as outlined in Section S.5, water’s density of 997 kg/m^3^ and a pressure of 1.01 × 10^5^ Pa, the bubble collapse time is estimated to be about 4 ns.

In principle, as outlined in the hypothesized sequence of events, we would expect the nucleation event to occur faster than the solute transport from the bubble interface back into the bulk solution. Based on Fickian diffusion, estimations as detailed in Section S.6. show that it would take 9 ns for the excess solute concentration at the bubble interface to be reduced by 50%. Thus, after the 6 ns laser pulse has ended, about half of the excess solute will remain at the bubble interface. Hence, we expect that the sequence of events that lead to bubble formation, growth and the subsequent pushing of solute molecules previously near the nanoparticle to the bubble interface would happen quickly enough for solute clustering and ordering leading to a nascent crystal before most of the solute can diffuse away from the concentrated layer into the bulk solution.

Comparing our results to the recent work by Korede et al.^24^ who also studied NPLIN in iron (II,III) oxide doped KCl solutions using microfluidics, but using two-phase slugs, suggests that the use of liquid–liquid slugs for KCl NPLIN is disadvantageous for several reasons. The extremely small size of the slugs (∼1.2 μL) means that only zero or one crystal can be observed in a given slug, so that *N*_crystal_ ≤ 1. This is because the nucleation rate is proportional to the sample volume, so the smaller the sample volume, the lower the nucleation rate at a given supersaturation.^36,30^ In contrast, in single-phase flow, one can create unlimited volume sizes and thus guarantee that *N*_crystal_ ≥ 1. Furthermore, the surface-to-volume ratio of the two-phase slugs is large, making heterogeneous nucleation much more probable, effectively adding more noise to the measurement of NPLIN probability. This background noise, referring to the nucleation yield without the laser, also skews the measured threshold intensities to larger values than would be obtained in the absence of this noise. In our study, we report no crystals when the laser was off and the supersaturated aqueous KCl solution moved through the capillary.

## 5 Conclusions

A capillary-based microfluidic system designed for NPLIN studies coupled with real-time microscopy was used to study NPLIN of doped aqueous KCl solutions. The microfluidic approach provided a better alternative to the labor-intensive batch approach for NPLIN experiments. The effect of nanoparticle concentration, supersaturation, laser intensity and filtration were studied. Filtration of the solution with 20 nm pore size suppressed crystal yield by an order of magnitude compared to unfiltered solutions. Dynamic Light Scattering and Zeta Potential measurements highlight the possibility of dense solute cluster shells around the nanoparticles in solution. Crystal size distribution results highlight the possibility to adopt the use of dopants with laser-induced nucleation to produce a narrow range of crystal sizes for industrial applications. A semiempirical model derived with respect to the impurity-heating hypothesis was created to explain the results. We also estimated the times needed for the solute molecules concentrated at the bubble interface to diffuse back into the bulk solution and found that during the 6 ns laser pulse most of the solute will remain at the concentrated layer at the bubble interface; thus, a crystal nucleation event should happen before the solute molecules diffuse away. The model exhibits a zero-laser intensity threshold, and experiments showed that nucleation could be observed as low as 1 MW/cm^2^. This is in contrast to previous findings of a threshold laser intensity in the range of 3–6 MW/cm^2^. No crystals were observed when the laser was off which implies that crystallization is induced by the laser. The results presented here highlight a significant step to a better quantitative understanding of NPLIN for solid crystals based on the colloidal impurity mechanism.

## Supporting Information Available

Figure with induced supersaturation vs cooled zone temperature, details on preparation of nanoparticle dispersion and doping of KCl solution, details of computational fluid dynamics simulation and infrared image of flow system, crystal yield experimental results table, estimation of nanobubble radius, and diffusion estimations (PDF)
