# Supplementary material for: Microfluidic Laser-Induced Nucleation of Iron (II,III) Oxide Nanoparticle-Doped Supersaturated Aqueous KCl Solutions
Source: Cryst Growth Des. 2024 Sep 28;24(20):8370–80. doi: 10.1021/acs.cgd.4c00885 (PMC11487495; doi:10.1021/acs.cgd.4c00885)
Supplement: Supplementary file 1 — cg4c00885_si_001.pdf [file cg4c00885_si_001.pdf]

SUPPORTING INFORMATION

Microfluidic Laser-Induced Nucleation of Iron (II,III) Oxide Nanoparticle-Doped Supersaturated Aqueous KCl solutions

Kelechi F. Ndukwe-Ajala, Jasmin M. Sabirin, Bruce A. Garetz*, and Ryan L. Hartman*

Department of Chemical and Biomolecular Engineering, NYU Tandon School of Engineering, Brooklyn, New York 11201, United States

S.1. Induced Supersaturation.

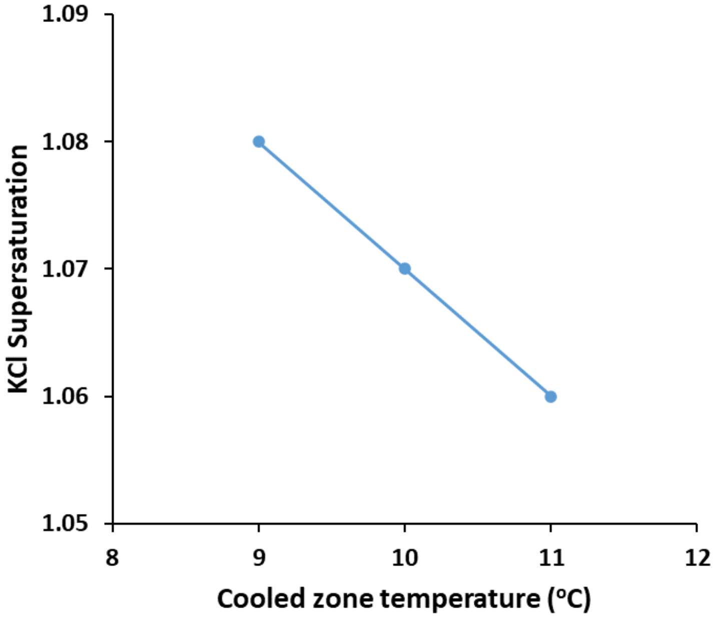

Figure S.1. Relationship between cooled zone temperature and KCl supersaturation

S.2. Preparation of nanoparticle dispersion.

A stock aqueous dispersion of nanoparticles was prepared by careful and clean dilution of the purchased concentration of 5mg/mL 30nm Fe_3O_4 nanoparticles dispersed in water. A concentration of 0.17 mg/mL was prepared by dilution with filtered (0.22μm pore) ultrapure water. In a clean vial, 685 μL of 5 mg/mL dispersion was added to 19.315mL filtered ultrapure water. The dispersion was sonicated for 1hr and stored at 6°C. The stock dispersion was kept at ambient temperature (~20°C) for 30 mins to reach room temperature and then sonicated for 30 mins before using to dope KCl solutions.

To dope the aqueous KCl solutions, the following calculations were done. For a 30nm diameter Fe_3O_4 particle, the volume of the spherical particle:

$$= \frac{4}{3}\pi r^3 = \frac{4}{3}\pi(15 \times 10^{-9} m)^3 = 1.4 \times 10^{-23} m^3 = 1.4 \times 10^{-17} mL$$

Using the density of Fe_3O_4 = 5.17 g/mL,[1] the mass of one particle = $7.2 \times 10^{-17} g$. For 6×10^9 Fe_3O_4 particles, the total mass = $4.3 \times 10^{-7} g$. Equivalently the nanoparticle number density of 6×10^9 Fe_3O_4 particles per mL solution can be expressed as $4.3 \times 10^{-7} g$ per mL solution.

Adding 120 μL of the stock 0.17 mg/mL Fe_3O_4 dispersion to 47 mL of the 3.89 M filtered KCl solution gives the nanoparticle concentration of $4.3 \times 10^{-7} g$ per mL solution.

$$= \frac{1.7 \times 10^{-4} \frac{g}{mL} \times 0.12mL}{47mL + 0.12mL} = 4.3 \times 10^{-7} \frac{g}{mL}$$

S.3. Computational Fluid Dynamics (CFD) simulation.

The CFD simulation was carried out using COMSOL Multiphysics® version 6.1 (Build: 357). Geometries were created with dimensions identical to those of the actual square capillary and thermoelectric devices. Borosilicate glass, ceramic, air, and aqueous KCl (at 25°C and 4.5 mol/kg) were assigned to the capillary tube, Peltier coolers, surrounding fluid, and fluid flowing through the capillary tube respectively. All materials except ceramic were available in the COMSOL material package. Ceramics properties used: thermal conductivity= 170 W/(m.K), density = 2607 kg/m^3, heat capacity at constant pressure = 850 J/(kg.K). The capillary and thermoelectric devices used a normal-sized mesh while the fluid used a finer mesh calibrated for fluid dynamics calculation.

Physics Interfaces and governing equations used in CFD

Heat Transfer in Solids and Fluids, and Laminar Flow interface was used to simulate the experimental conditions. Under the Heat Transfer in Solids and Fluids interface, the governing equation comes from heat balances and is shown as follows:

$$\rho C_p \boldsymbol{u} \cdot \nabla T + \nabla \cdot \boldsymbol{q} = Q + Q_{ted}$$

$$\boldsymbol{q} = -k \nabla T$$

where ρ is the density [kg/m^3], C_p is the specific heat capacity at constant stress [J/(kg.K)], u is the fluid velocity vector [m/s], T is temperature [K], $\boldsymbol{q}$ is the heat flux by conduction [W/m^2], Q is heat source/sink [W/m^3], Q_{ted} is the heat source accounting for thermoelastic damping [W/m^3] and k is thermal conductivity [W/(m.K)]. The above equation applies to the two Peltier cooler blocks.

The governing equation for the heat transfer within the fluid itself is shown below:

$$\rho C_p \boldsymbol{u} \cdot \nabla T + \nabla \cdot \boldsymbol{q} = Q + Q_p + Q_{vd}$$

$$\boldsymbol{q} = -k\nabla T$$

where Q_p is the heat source accounting for the work done by pressure changes [W/m^3], Q_{vd} is the heat source accounting for viscous dissipation in the fluid [W/m^3].

The study was conducted for steady state. The ambient temperature of the system was set to 293.15K. For laminar flow, the single-phase fluid flow interface is based on the Navier-Stokes equations and the governing equations include:

$$p(\boldsymbol{u} \cdot \nabla)\boldsymbol{u} = \nabla \cdot [-p\boldsymbol{I} + \boldsymbol{K}] + \boldsymbol{F}$$

$$\rho \nabla \cdot \boldsymbol{u} = 0$$

where $\boldsymbol{u}$ is the fluid velocity vector [m/s], $\boldsymbol{I}$ is the Identity matrix [unitless], $\boldsymbol{K}$ is the viscous stress tensor [Pa] and $\boldsymbol{F}$ is the volume force vector [N/m^3].

The fluid is incompressible and is entering at 293.15K (ambient temperature). The no-slip boundary condition was assumed at the wall. Initial fluid velocity was set to zero. The mass flow rate of the fluid was set to 5.1×10^{-6} kg/s (300 μL/min).

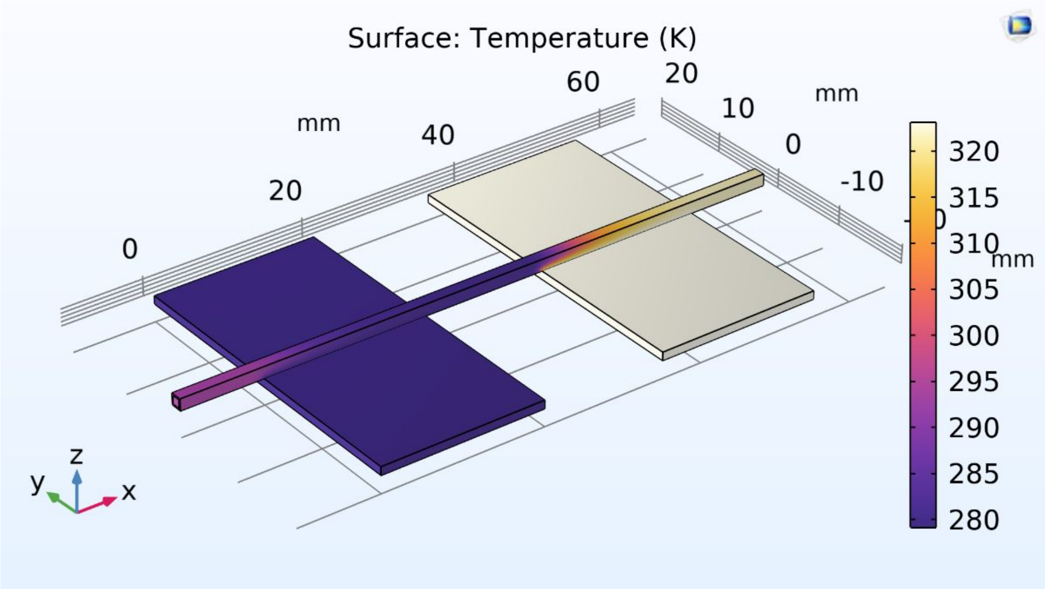

Figure S.2. CFD simulation temperature map result

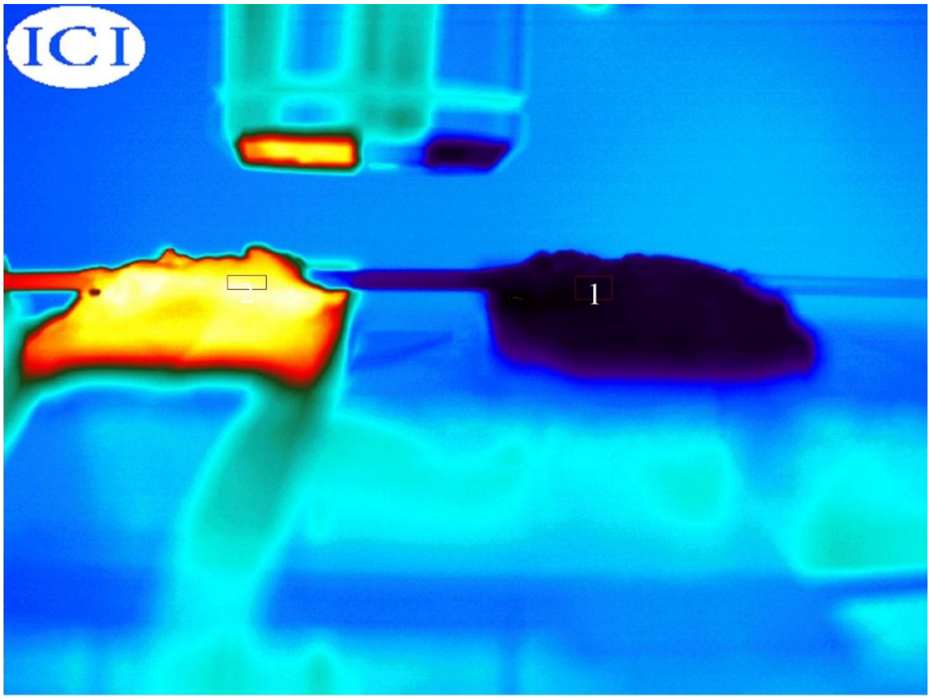

Figure S.3. The temperature color map of the capillary setup. Zone 1 (cold Peltier region) has an average temperature of 10.92 $\pm$ 0.24 ^{o}C and Zone 2 (hot Peltier region) has an average temperature of 41.36 $\pm$ 0.54 ^{o}C

S.4. Crystal yield results.

Supersaturation/Nanoparticle number concentration												
1.06/ 6E+9 per mL	**1**	**2**	**4**	**6**	**8**	**10**	**12**	**15**	**18**	**20**	Laser intensity (MW/cm²)	
	8	8	20	15	26	27	31	34	26	33	run 1	Number of crystals observed
	4	10	21	18	27	41	24	35	33	21	run 2	
	5	11	19	18	26	13	32	33	25	23	run 3	
	0.5	0.5	0.5	1	1	0.5	0.5	0.5	0.25	0.25	Video time (min)	
	0.15	0.15	0.15	0.3	0.3	0.15	0.15	0.15	0.075	0.075	Irradiated solution volume (mL)	
	37.78	64.44	133.33	56.67	87.78	180	193.33	226.67	373.33	342.22	Average number of crystals normalized to 1 mL	
	11.33	8.31	5.44	4.71	1.57	76.21	23.73	5.44	47.45	69.99	Standard deviation	
1.07/ 6E+9 per mL	**1**	**2**	**4**	**6**	**8**	**10**	**12**	**15**	**18**	**20**	Laser intensity (MW/cm²)	
	4	12	18	31	59	65	54	80	32	50	run 1	Number of crystals observed
	5	20	12	28	49	44	55	86	40	61	run 2	
	5	18	22	37	60	40	53	80	70	47	run 3	
	0.5	0.5	0.5	1	1	0.5	0.5	0.5	0.25	0.25	Video time (min)	
	0.15	0.15	0.15	0.3	0.3	0.15	0.15	0.15	0.075	0.075	Irradiated solution volume (mL)	
	31.11	111.11	115.56	106.67	186.67	331.11	360.00	546.67	631.11	702.22	Average number of crystals normalized to 1 mL	
	3.14	22.66	27.40	12.47	16.56	73.10	5.44	18.86	218.10	80.25	Standard deviation	

**Supersaturation/Nanoparticle number concentration**												
1.08/ 6E+9 per mL	**1**	**2**	**4**	**6**	**8**	**10**	**12**	**15**	**18**	**20**	Laser intensity (MW/cm^2)	
	10	33	72	63	81	51	37	72	88	97	run 1	Number of crystals observed
	9	38	71	81	109	79	42	61	68	82	run 2	
	12	35	71	84	99	72	46	71	84	105	run 3	
	0.5	0.5	0.5	0.5	0.5	0.5	0.25	0.25	0.25	0.25	Video time (min)	
	0.15	0.15	0.15	0.15	0.15	0.15	0.075	0.075	0.075	0.075	Irradiated solution volume (mL)	
	68.89	235.56	475.56	506.67	642.22	448.89	555.56	906.67	1066.67	1262.22	Average number of crystals normalized to 1 mL	
	8.31	13.70	3.14	61.82	77.24	79.32	49.09	66.22	115.21	127.11	Standard deviation	
1.06/ 3E+9 per mL	**1**	**2**	**4**	**6**	**8**	**10**	**12**	**15**	**18**	**20**	Laser intensity (MW/cm^2)	
	5	10	3	6	13	12	15	18	22	22	run 1	Number of crystals observed
	6	4	4	12	13	16	10	24	29	9	run 2	
	5	2	4	10	8	12	13	13	17	20	run 3	
	4	5	2	2	1	1	1	1	1	1	Video time (min)	
	1.2	1.5	0.6	0.6	0.3	0.3	0.3	0.3	0.3	0.3	Irradiated solution volume (mL)	
	4.44	3.56	6.11	15.56	37.78	44.44	42.22	61.11	75.56	56.67	Average number of crystals normalized to 1 mL	
	0.39	2.27	0.79	4.16	7.86	6.29	6.85	14.99	16.41	19.05	Standard deviation	

Table S.1. Laser-Induced nucleation experimental results for different supersaturation, nanoparticle number concentrations and laser intensities.

S.5. Estimation of Nanobubble radius

The methodology to estimate the size of the bubble formed is the same as the approach utilized by Ward et al.[2]

The nanoparticles are iron (II,III) oxide and spherical. The surrounding liquid is assumed to be water (the mole fraction of KCl used in this study is calculated to be 7.7 x 10^{-2}. The energy absorbed by a spherical particle of radius a is given as:

$$E_{abs} = \pi a^2 Q_{abs}(a) I t_p \tag{1}$$

where Q_{abs} is the absorption efficiency. Using MiePlot v4.6.21 (written by Philip Laven), Mie scattering calculations were done to determine Q_{abs}. The complex refractive index of iron (II,III) oxide at 1064nm is n = 2.112 + 0.3698i.[3] The particle is surrounded by water and for a = 15nm, we obtain Q_{abs} = 0.061. At a laser intensity I = 1 MW/cm^2 for a single laser pulse duration t_p= 6 ns, the energy absorbed by the particle E_{abs} = 2.59 x 10^{-15} J.

All of the absorbed energy is assumed to rapidly transfer to the surrounding shell of water hence instantaneously vaporizing it. Water explosively boils at about 80% of the critical temperature ($T_k \sim 0.8T_{cr}$, where T_{cr} = 647.1 K)[4,5,6] . The number of moles of water, n can be estimated from:

$$E_{abs} = n\,(\Delta_{vap}U_{T_1} + \int_{T_0}^{T_1} c_v(T)dT) \tag{2}$$

where T_0 = solution temperature (for calculations here T_0 = 11°C = 284 K), $T_1 = T_k$ = 520 K, and c_v is the constant-volume heat capacity of liquid water. Water's heat capacity data[7] was estimated with a polynomial fit and used to calculate the integral. The internal energy change for vaporization was approximated by $\Delta_{vap}U_{T_1} = \Delta_{vap}H_{T_1} - RT_1$, where the enthalpy of vaporization of water $\Delta_{vap}H_{T_1}$ = 31 kJ/mol.[8] We calculate n = 5.3 x 10^{-20} mol and V_1 = 9.6 x 10^{-25} m^3 at the density ρ_0 (T_0) = 999.56 kg/m^3.[9]

Assuming reversible isentropic adiabatic expansion, the final bubble size can be calculated. Assuming an ideal case, PV^{χ} = constant, where $\chi = c_p/c_v$ is the ratio of heat capacities; for water at 284 K, χ = 1.328. The initial pressure of the water vapor is estimated to be $P_1 = \frac{nRT_1}{V_1}$ = 239.8 MPa. Assuming a final pressure equal to ambient pressure, P_2 = 101.3 kPa, we calculate the final volume of the expanded water, V_2 = 3.3 x 10^{-22} m^3. Combined with the volume of the particle, this gives a final bubble radius of r_2 = 43 nm. Ward et al. 2015[2] compared the result of the non-ideal case and the results were very close with the ideal case.

S.6. Diffusion Estimations

For a 30nm Fe_3O_4 nanoparticle exposed to 1 MW/cm^2 and initially at T =11°C (S=1.06), the final bubble radius is estimated to be 43 nm. We can perform calculations to estimate the concentration of KCl at the bubble interface.

Volume of shell with prior KCl solution:

$$= \frac{4}{3}\pi\left(r_b^3 - r_{np}^3\right) = \frac{4}{3}\pi\left((43nm)^3 - (15nm)^3\right) = 3.2 \times 10^5 nm^3 = 3.2 \times 10^{-16}\, mL$$

Concentration of KCl in bulk aqueous solution = 0.34 g KCl in 1 g water. Using densities of solid KCl and water = 1.98 g/mL and 0.997 g/mL respectively. We can express the concentration of KCl with volumes = 0.172 mL KCl and 1.003 mL water. And assuming additive volumes, we can estimate the mass of KCl in shell:

$$= \frac{0.172\, mL}{0.172\, mL + 1.003\, mL} \times 3.2 \times 10^{-16}\, mL \times 1.98\, \frac{g}{mL} = 9.3 \times 10^{-17}\, g\, KCl$$

KCl concentration in bulk:

$$= \frac{0.34g}{0.172mL + 1.003mL} = 0.29\, g\, KCl\, per\, mL\, solution$$

We can estimate the KCl concentration at a given time and distance from the concentrated layer using this equation based on Fickian diffusion[10]:

$$C(x,t) = \frac{M}{\sqrt{4\pi Dt}} \exp\left(-\frac{x^2}{4Dt}\right) \tag{3}$$

where x is the diffusing distance, t is time, D is the diffusion coefficient for KCl, M is the mass of solute per unit area. The $C(x,t)$ distribution is a Gaussian function.

We adapt equation 3 for our problem to account for the background KCl concentration,

$$C(x,t) = \frac{M}{\sqrt{4\pi Dt}} \exp\left(-\frac{x^2}{4Dt}\right) + C_b = C_{excess} + C_b \tag{4}$$

where C_b is the KCl concentration in the bulk solution = 0.29 g/mL and C_{excess} is the excess solute concentration available for diffusion.

We define C_{solid} = 1.98 g/mL as the density of solid KCl. However, we assume C_{peak}= 1 g/mL ($\approx \frac{1}{2}C_{solid}$) and the ratio of 3.4 between C_b and C_{peak} highlights an extreme condition that could result in spontaneous nucleation for aqueous KCl solutions. However, for our estimations we assume C_{peak}= 1 g/mL for the solute concentration in the concentrated layer at the bubble interface.

To estimate the thickness of the concentrated solute layer at the bubble interface, we assume a maximum density of solute in that layer equal to C_{peak}. Also, we assume the thickness of the layer is very small compared to the bubble diameter.

$$C_{peak} = \frac{Mass}{Volume} = \frac{m}{A \times t_l} \rightarrow t_l = \frac{m}{A \times C_{peak}} \tag{5}$$

where m is the mass of KCl previously inside the shell, A is the surface area of the bubble, t_l is the layer thickness at the bubble interface.

$$t_l = \frac{9.3 \times 10^{-17}\, g}{4\pi(43 \times 10^{-9} m)^2 \times 1 \frac{g}{mL} \times \frac{1mL}{10^{-6} m^3}} = 4.0\, nm$$

For equation 3, the spreading induced by diffusion can be represented by σ, the standard deviation of a Gaussian distribution [10]:

$$\sigma = \sqrt{2Dt} \tag{6}$$

and for a Gaussian function[11], the full width at half maximum (FWHM): FWHM = 2.35σ. For our estimations, we assume the layer thickness is equal to the half width at half maximum (HWHM) for the Gaussian function. Thus, HWHM = 1.17σ.

$$\sigma_1 = \frac{4.0\, nm}{1.17} = 3.4\, nm$$

We would like to estimate the time t_1, required for $C(x,t)$ in equation 3 to exhibit a σ_1 value of 3.4 nm. Diffusion coefficient of KCl in aqueous solution[12] at 20°C for 1.0 M = $1.891 \times 10^{-9}\, \frac{m^2}{s}$.

$$t_1 = \frac{\sigma_1^2}{2D} = \frac{(3.4nm)^2 \times \dfrac{10^{-18} m^2}{1\, nm^2}}{2 \times 1.891 \times 10^{-9}\, \dfrac{m^2}{s}} = 3.1\, ns \approx 3\, ns$$

From equation 4,

$$C_{excess} = \frac{M}{\sqrt{4\pi Dt}} \exp\left(-\frac{x^2}{4Dt}\right) \quad (7)$$

The peak of C_{excess} is at x = 0 and at two different times, we have this relation:

$$\frac{C_{excess}(0,t_2)}{C_{excess}(0,t_1)} = \left(\frac{t_1}{t_2}\right)^{\frac{1}{2}} \quad (8)$$

Time for C_{excess} to be reduced by 50%

$$t_2 = \frac{t_1}{(0.5)^2} = \frac{3\, ns}{0.25} = 12\, ns$$

$$\Delta t = t_2 - t_1 = 9\, ns$$

Thus, it would take 9 ns for the excess solute concentration to be reduced by 50%.

REFERENCES

(1) *Density of Magnetite in 285 units and reference information.* https://www.aqua-calc.com/page/density-table/substance/magnetite (accessed 2024-06-24).

(2) Ward, M. R.; Jamieson, W. J.; Leckey, C. A.; Alexander, A. J. Laser-Induced Nucleation of Carbon Dioxide Bubbles. *J. Chem. Phys.* **2015**, *142* (14), 144501. https://doi.org/10.1063/1.4917022.

(3) Polyanskiy, M. N. Refractiveindex.Info Database of Optical Constants. *Sci Data* **2024**, *11* (1), 94. https://doi.org/10.1038/s41597-023-02898-2.

(4) Dou, Y.; Zhigilei, L. V.; Winograd, N.; Garrison, B. J. Explosive Boiling of Water Films Adjacent to Heated Surfaces: A Microscopic Description. *J. Phys. Chem. A* **2001**, *105* (12), 2748–2755. https://doi.org/10.1021/jp003913o.

(5) Avedisian, C. T. The Homogeneous Nucleation Limits of Liquids. *Journal of Physical and Chemical Reference Data* **1985**, *14* (3), 695–729. https://doi.org/10.1063/1.555734.

(6) Lubetkin, S. D. Why Is It Much Easier To Nucleate Gas Bubbles than Theory Predicts? *Langmuir* **2003**, *19* (7), 2575–2587. https://doi.org/10.1021/la0266381.

(7) Wagner, W.; Pruß, A. The IAPWS Formulation 1995 for the Thermodynamic Properties of Ordinary Water Substance for General and Scientific Use. *Journal of Physical and Chemical Reference Data* **2002**, *31* (2), 387–535. https://doi.org/10.1063/1.1461829.

(8) Lide, D. R.; Baysinger, G.; Chemistry, S.; Berger, L. I.; Goldberg, R. N.; Kehiaian, H. V. CRC Handbook of Chemistry and Physics.

(9) *Water - Density, Specific Weight and Thermal Expansion Coefficients.* https://www.engineeringtoolbox.com/water-density-specific-weight-d_595.html (accessed 2024-05-14).

(10) Chapter 2:DIFFUSION. https://cushman.host.dartmouth.edu/courses/engs43/Chapter2.pdf (accessed 2024-06-21).

(11) *PSF (GNU Astronomy Utilities).* https://www.gnu.org/software/gnuastro/manual/html_node/PSF.html (accessed 2024-06-24).

(12) Lobo, V. M. M.; Ribeiro, A. C. F.; Verissimo, L. M. P. Diffusion Coefficients in Aqueous Solutions of Potassium Chloride at High and Low Concentrations. *Journal of Molecular Liquids* **1998**, *78* (1), 139–149. https://doi.org/10.1016/S0167-7322(98)00088-9.
